# Off‐label prescribing of targeted anticancer therapy at a large pediatric cancer center

**DOI:** 10.1002/cam4.3349

**Published:** 2020-08-04

**Authors:** Mir Lim, David S. Shulman, Holly Roberts, Anran Li, Jessica Clymer, Kira Bona, Hasan Al‐Sayegh, Clement Ma, Steven G. DuBois

**Affiliations:** ^1^ Boston University School of Medicine Boston MA USA; ^2^ Dana‐Farber/Boston Children's Cancer and Blood Disorders Center Harvard Medical School Boston MA USA

**Keywords:** dosing, neuro‐oncology, off‐label drug, pediatric cancer, target therapy, toxicity

## Abstract

**Background:**

Off‐label drug prescribing is common in pediatric clinical medicine, though the extent and impact of this practice in pediatric oncology has not yet been characterized.

**Methods:**

We completed a retrospective single‐institution cohort study evaluating prevalence, characteristics, and clinical outcomes of off‐label prescribing of 108 FDA‐approved targeted anticancer drugs in patients < 30 years old treated for cancer from 2007 to 2017. Dosing strategies were adjusted for body size and compared to FDA‐approved adult dosing regimen. A composite toxicity endpoint was defined as a patient having unplanned clinic visits, emergency department visits, or unplanned hospital admissions that were at least possibly related to the off‐label treatment.

**Results:**

The overall prevalence of off‐label use of targeted therapies was 9.2% (n = 374 patients). The prevalence increased significantly over the study period (*P* < .0001). Patients treated off‐label were more likely to have neuro‐oncology diagnoses compared to patients not treated off‐label (46% vs 29%; *P* < .0001). Of the 108 potential agents, 38 (35%) were used by at least one patient. The median starting dose was below the FDA‐approved normalized dose for 44.4% of agents. Fifteen percent of patients had a complete response while receiving off‐label therapy, 38% experienced toxicity as defined, and 13% discontinued off‐label therapy due to toxicity.

**Conclusions:**

In this real‐world evaluation of prescribing at a large pediatric cancer center, off‐label prescribing of FDA‐approved targeted therapies was common, increasing in prevalence, encompassed a broad sample of targeted agents, and was tolerable. Clinicians commonly start dosing below the equivalent FDA‐approved dose.

## Graphical abstract

Off‐label prescribing patterns and outcomes with targeted anticancer agents have not been previously described in pediatric oncology. In this analysis from a large pediatric cancer center, off‐label prescribing of FDA‐approved targeted therapies was common, increasing, encompassed a broad sample of targeted agents, and was tolerable.

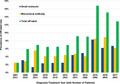

## 1 INTRODUCTION

Off‐label drug prescribing in the United States refers to the application of an FDA‐approved drug beyond the approved label and may include an alternative indication, unapproved patient age, or alternative dosing or duration of treatment.^1^ Off‐label prescribing is used in the absence of an approved treatment, or when an approved standard therapy is available but perceived efficacy, toxicity, and quality of life considerations may favor the use of an off‐label medication rather than an approved treatment.^2^ Off‐label prescribing is common across all pediatric disciplines, though may be enriched in specific populations.^3^ For example, European studies have shown that at least one‐third of children in hospitals and up to 90% of neonates in a neonatal intensive care unit receive off‐label therapies.^4^ Similarly, off‐label prescribing is common within the field of oncology.^5^

Most conventional cytotoxic chemotherapy agents used to treat children with cancer do not carry a pediatric label indication. Over the last 20 years, more than 100 targeted anticancer agents have been approved to treat various oncologic diagnoses,^6^ and most of these also do not carry a pediatric label indication. Recent studies have evaluated the prevalence of off‐label prescribing in adults with cancer. One study found that, of the 10 most commonly prescribed intravenous cancer drugs, 30% of the use was off‐label prescriptions.^7^ Little is known about the patterns of off‐label prescribing of targeted anticancer therapies in the context of pediatric oncology, though several factors may drive this practice. The rarity of pediatric cancer has resulted in an overall dearth of approved targeted therapies, and the poor outcomes for patients with relapsed disease likely increases demand. The impact of off‐label use on children with cancer in the era of targeted therapy is not known, including resultant toxicity and antitumor activity.

To fill these gaps in our knowledge, we aimed to describe the prevalence of off‐label prescribing of targeted anticancer agents in children and young adults with cancer and to describe the types of off‐label targeted agents used most often in this population. We also sought to determine whether clinical or demographic features were associated with receipt of off‐label targeted therapy. Finally, we obtained data on the dosing strategies utilized for off‐label prescribing and clinical outcomes following off‐label use of targeted agents in this population.

## 2 PATIENTS AND METHODS

### 2.1 Design and patient population

We conducted a retrospective cohort study of patients treated for cancer with targeted therapies at Dana‐Farber/Boston Children's Cancer and Blood Disorders Center between January 1, 2007 and December 31, 2017. The start date was chosen to align with the start of the first full year of the use of the current Boston Children's Hospital electronic medical record.

We identified patients <30 years of age who received a targeted, off‐label anticancer therapy. We utilized a list of all targeted anti‐cancer agents approved by the FDA between 1997 and 2017, excluding FDA‐approved cytotoxic chemotherapy drugs (Table S1). We searched multiple institutional databases using Dana‐Farber Cancer Institute outpatient pharmacy records and Boston Children's Hospital outpatient and inpatient medication lists to identify patients who received any of the targeted agents of interest while under the care of a Dana‐Farber/Boston Children's oncologist. A patient was coded as having received that agent on an off‐label basis if the FDA label at the time they started the therapy did not specify use in children or use in their disease. The following types of patients were not considered to have received off‐label therapy: (a) receipt as part of a clinical trial or prescribed as part of a compassionate use protocol; (b) receipt of an agent (eg, rituximab) for a non‐oncologic indication (eg, autoimmune disorder); and (c) receipt of an agent with the appropriate pediatric label indication, but following a different dosing regimen or duration.

To identify a control group of patients for the purposes of calculating the prevalence of off‐label prescribing and to identify demographic and clinical factors associated with the use of off‐label prescribing, we selected patients who were diagnosed between January 1, 2007 and December 31, 2017 and were younger than 30 years of age at the time of diagnosis, using the Boston Children's Hospital Tumor Registry.

Retrospective review of patient data for this analysis was approved by the Dana‐Farber Institutional Review Board (IRB) in accordance with US Federal Policy for the Protection of Human Subjects.

### 2.2 Variables

We performed a detailed review of the electronic medical record to capture additional data for patients who met the defined criteria of having received an off‐label targeted anticancer drug. We collected information regarding the number and types of off‐label targeted agents used by each patient, diagnosis, stage of cancer, and previously administered therapies. We also recorded the race, ethnicity, sex, age, type of insurance, and ZIP code of each patient. We classified insurance as public or private. ZIP codes were categorized as high‐poverty neighborhoods (≥20% of persons living below 100% federal poverty level (FPL)) or low‐poverty neighborhoods (<20% of persons below 100% FPL) in concert with the US Census definitions and prior literature.^8^, ^9^, ^10^

For each episode of off‐label use, we also collected dosing strategy, the duration of therapy, as well as whether the patient had a complete response as assessed by the treating clinician. For comparison of dosing in our cohort and the FDA‐approved dose, we normalized to dose/m^2^ assuming adult body surface area of 1.7 m^2^. For agents with multiple approved dosing regimens, we compared the most relevant approved dosing strategy. Detailed capture of all adverse events while receiving an off‐label agent was outside the scope of this analysis. As a proxy for understanding clinically meaningful toxicity, the following events during off‐label treatment were defined as meeting toxicity criteria for this analysis: any unplanned clinic visits, emergency department visits, or unplanned hospital admissions that were deemed at least possibly related to the off‐label therapy. Unplanned clinic visits were defined as those not scheduled for planned cancer‐directed therapy administration based on the patient's defined treatment protocol. For example, a patient coming in for blood product transfusion in between treatment cycles would be marked as an unplanned clinic visit since the patient had to receive additional treatment to manage a toxicity. We separately recorded any episodes of dose reduction or early discontinuation of the off‐label targeted therapy from medical record abstraction.

For the control group, we extracted available clinical and demographic data (age at diagnosis, sex, ZIP code, and disease category) using an internal tool (the Pediatric Patient Informatics Platform, or PPIP) that harmonizes patient data from a range of Dana‐Farber and Boston Children's source systems.

### 2.3 Statistical analysis

The overall prevalence of off‐label use was calculated by dividing the number of unique patients treated at least once with off‐label targeted therapy by the overall number of unique pediatric oncology patients who were initially diagnosed or initially treated by off‐label therapy between 2007 and 2017. For the purpose of this analysis, patients who received off‐label therapy were counted in the year they received their first off‐label therapy. Patients who did not receive off‐label therapy were counted in the year of their initial diagnosis. In addition, a sensitivity analysis was completed to determine the prevalence of off‐label use among only those patients who received systemic therapy [cases and controls with at least one treatment plan recorded in the computerized chemotherapy order entry (COE2) system] between 2012 and 2017 (date range with available data), thereby excluding those who received only local interventions or observation.

Descriptive statistics (frequencies and proportions) were used to summarize the clinical and demographic characteristics of the cohort stratified by off‐label targeted therapy. Clinical and demographic features were compared using chi‐squared or Fisher exact test for categorical variables and Wilcoxon rank‐sum for continuous variables. Changes in prevalence of off‐label use over time were assessed using logistic regression with year of off‐label use as a continuous predictor. Dosing strategies, duration of treatment and toxicity were analyzed descriptively. Statistical analysis was performed in SAS version 9.4 (SAS Institute Inc). Two‐sided *P*‐values < .05 were considered statistically significant.

## 3 RESULTS

### 3.1 Prevalence of use of off‐label targeted therapies

**FIGURE 1:**
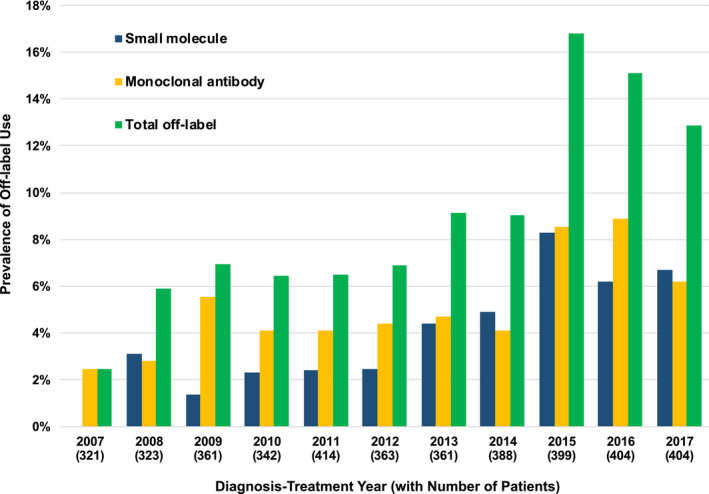
Prevalence of off‐label use by diagnosis‐treatment year and agent type for patients diagnosed or treated between 2007 and 2017. For the purpose of this analysis, patients who received off‐label therapy were counted in the year they received their first off‐label therapy. Patients who did not receive off‐label therapy were counted in the year of their initial diagnosis. Numbers in parentheses indicate number of patients included in denominator for each yearly prevalence calculation

Our search yielded 748 potential patients treated off‐label with at least one of the 108 targeted therapies included in the search. Of these, we identified 374 unique patients who met our definition of off‐label use of a targeted therapy for cancer diagnoses during the study period. We identified 3706 control patients initially diagnosed during the study period, for a total study population of 4080 patients. The prevalence of off‐label use over the entire study period was 9.2% (95% confidence interval [CI], 8.3%‐10.1%). The prevalence of off‐label use increased significantly over the study period. For each increasing year from 2007 to 2017, the odds ratio for receiving off‐label targeted therapy in that year compared to the preceding year was 1.16 (95% CI: 1.12‐1.2; *P* < .0001; Figure 1). The overall prevalence of off‐label use was 22.3% (n/N = 168/753; 95% CI, 19.3%‐25.5%) when we restricted the population of interest to those cases and controls with a treatment plan for systemic therapy between 2012 and 2017 in the computerized drug order entry system, thereby excluding patients who received only local interventions or observation.

### 3.2 Characteristics of patients treated with off‐label targeted therapies

Characteristics of patients treated with off‐label targeted therapies are presented in Table 1. Among the 374 patients treated with an off‐label targeted therapy, there were 571 instances of off‐label use, for an average of 1.5 off‐label agents used per patient in the cohort. The pattern of off‐label use was significantly different among disease categories (*P* < .0001). Neuro‐oncology diagnoses were more common among patients treated with off‐label targeted therapies (46%) compared to patients not treated with off‐label therapies (29%). Age at diagnosis was not statistically significantly different in patients treated with off‐label therapy compared to the control group (*P* = .2). There were no statistically significant differences in patient sex or area‐based poverty between patients treated with and without off‐label targeted therapies.

**TABLE 1:** Patient characteristics

	Off‐label use N = 374 n/N (%) or median (range)	No off‐label use N = 3706 n/N (%) or median (range)	*P*‐value (*χ* ^2^, Fisher exact, or Wilcoxon rank‐sum test)
Age at diagnosis (y)	10 (0.1, 25.9)	9 (0, 29.1)	.2
Sex			.07
Male	222/374 (59)	2018/3706 (54)	
Female	152/374 (41)	1688/3706 (46)	
Disease category			<.0001
Heme malignancy	96/374 (26)	1229/3629 (34)	
Solid tumor	106/374 (28)	1275/3629 (35)	
Neuro‐oncology	172/374 (46)	1047/3629 (29)	
Other	0/374 (0)	78/3629 (2)	
Area‐level poverty			.6
High poverty (≥20% below federal poverty level)	40/342 (12)	432/3414 (13)	
Low poverty (<20% below federal poverty level)	302/342 (88)	2982/3414 (87)	
Insurance			
Private	184/313 (59)		
Public	129/313 (41)		
Stage at diagnosis			
Localized	209/316 (66)		
Metastatic	107/316 (34)		
Stage at first off‐label therapy			
Localized	160/316 (51)		
Metastatic	156/316 (49)		
Number of treatment lines prior to first off‐label use			
0	87/374 (23)		
1	124/374 (33)		
2	75/374 (20)		
3 or more	88/374 (24)		
Prior radiation before the start of off‐label therapy	168 (45)		
Prior allogeneic stem cell transplant before the start of the first off‐label therapy	41 (8)		

Only 23% of patients treated with off‐label targeted therapies received them as part of their frontline therapy. Of these patients who received off‐label therapy as part of frontline therapy, the most common diagnoses included lymphoma/post‐transplant lymphoproliferative disorder (n = 48) and glioma (n = 15). In addition, 33% of patients had received one line of prior treatment, 20% had received two prior treatment lines, and 24% had received three or more prior lines of treatment.

### 3.3 Broad range of targeted therapies used off‐label

We next evaluated the types of agents and regimens being used off‐label. Of the 108 FDA‐approved targeted therapies included in this analysis, 38 agents (35%) were used off‐label by at least one patient. Off‐label use was divided between small molecule and monoclonal antibody agents, with 54% of off‐label uses involving small molecule agents and 46% monoclonal antibody agents (Table 2). The three most commonly used off‐label small molecule agents were thalidomide (n = 59 patients), sorafenib (n = 52), and everolimus (n = 38). The three most commonly used off‐label monoclonal antibody agents were bevacizumab (n = 156), rituximab (n = 59), and pembrolizumab (n = 17; Table S2). In 56% of cases, off‐label agents were given with conventional chemotherapy. In 23% of cases, off‐label agents were given with other targeted therapies.

**TABLE 2:** Details of off‐label prescribing patterns for 571 distinct uses

		Type of off‐label drug	
	Overall N = 571 n (%)	Small Molecule N = 308 n (%)	Monoclonal Antibody N = 263 n (%)
Duration of off‐label use (Median and range)	99 d (1, 3412) n = 544	93 d (1, 3412) n = 285	104 d (1, 3063) n = 259
Dose modified	132/562 (23%)	113/302 (37%)	19/260 (7%)
Type of modification			
Decreased	71/132 (54%)	56/113 (50%)	15/19 (79%)
Increased	61/132 (46%)	57/113 (50%)	4/19 (21%)
Given with conventional chemotherapy	322/570 (56%)	132/307 (43%)	190/263 (72%)
Given with other targeted therapy	129/571 (23%)	68/308 (22%)	61/263 (23%)

### 3.4 Dosing strategies for off‐label targeted therapies

The median starting dose for each off‐label drug is compared to the normalized FDA approved dose in Table S1. In 44.4% (n = 16), 22.3% (n = 8), and 33.3% (n = 12) of 36 drugs used off‐label, the median starting dose was less than, greater than, or equal (within 10%) to the normalized FDA‐approved dose, respectively. Two agents were administered topically and dose comparisons were not performed.

Overall, dose modifications were made in 23% (132/562) of instances of off‐label therapy (Table 2). In patients who had dose modifications, 46% (61/132) of the modifications were dose increases. Dose modifications were less common in instances of off‐label monoclonal antibody use (19/260; 7%) compared to small molecule inhibitors (113/302; 37%; *P* < .001). If modified, monoclonal antibody agents had dose reductions in 79% (15/19) of instances, while small molecules had dose reductions in 50% (56/113).

### 3.5 Efficacy and toxicity endpoints

The median duration of off‐label use was 99 days with a broad range (1‐3412 days; Table 2). Nine agents had a median duration of use greater than 100 days (Table S2). All patients included in response assessments had active disease to follow prior to the start of off‐label therapy, and patients who started off‐label therapy while in remission were excluded from these calculations. Overall, 15% (80/544) of instances of off‐label use yielded a complete response when given as monotherapy or as a component of combination therapy (Table 3). Complete responses were achieved in 19% (49/255) of instances of monoclonal antibody use and in 11% (31/289) of instances of small molecule use. Focusing exclusively on use of off‐label therapies without concomitant chemotherapy, 12% (20/174) of instances yielded complete responses (Table S3). At the end of the study period, 97% of off‐label therapies had been discontinued, with the only ongoing therapy in 14 patients who received off‐label small molecules. For all types of off‐label therapy, the most common reason (46% of instances) for stopping therapy was progression of disease. Disease progression leading to discontinuation occurred in 39% of instances of monoclonal antibody use compared to 52% of small molecule use.

**TABLE 3:** Response and toxicity for 571 distinct off‐label uses

		Type of Off‐Label Drug	
	Overall N = 571 (n/N, %)	Small Molecule N = 308 (n/N, %)	Monoclonal Antibody N = 263 (n/N, %)
Complete response	80/544 (15%)	31/289 (11%)	49/255 (19%)
Met toxicity criteria^a^	208/558 (38%)	118/295 (40%)	90/257 (35%)
Drug discontinued at time of data collection	543/544 (97%)	285/299 (95%)	258/259 (100%)
Reason for stopping off‐label therapy			
Progression	249/543 (46%)	148/285 (52%)	101/258 (39%)
Other	138/543 (25%)	68/285 (24%)	70/258 (27%)
Completed planned cycles	85/543 (16%)	24/285 (8%)	61/258 (24%)
Toxicity	71/543 (13%)	45/285 (16%)	26/258 (10%)

^a^ Unplanned clinic visits for toxicity, emergency department visits for toxicity, and/or unplanned admissions for toxicity.

Using our composite toxicity endpoint, 38% of instances of off‐label use met criteria for toxicity. In 50% (103/208) of instances with toxicity, patients were receiving off‐label therapy in combination with conventional chemotherapy or other targeted agents at the time toxicity occurred. In the small molecule therapy category, 40% of instances were associated with toxicity, and 16% of patients discontinued therapy due to toxicity. Similarly, 35% of instances of monoclonal antibody use were associated with toxicity (*P* = .3 for comparison with small molecule inhibitor toxicity rates). However, only 10% of patients treated with monoclonal antibodies discontinued therapy due to toxicity, fewer than among those treated with small molecule inhibitors (*P* = .055; Table 3). In addition to the defined toxicity criteria, 13% of instances of off‐label use resulted in dose reduction of the off‐label targeted therapy.

## 4 DISCUSSION

We provide a comprehensive assessment of off‐label use of targeted anticancer agents in a large pediatric academic medical center. We found an increasing prevalence of off‐label use during the study period, with an overall prevalence of 9.2% among all patients in our study. When restricting the analytic cohort to patients with a treatment plan for systemic anticancer therapy, the prevalence rate was 22.3%. It is not clear if the increasing prevalence over time reflects greater availability of FDA‐approved targeted therapies, changes in clinical practice (eg, genomic profiling), or some combination of these factors. Although some patients received off‐label targeted agents as part of frontline therapy, most received this as therapy for relapsed disease. In this study, 38 different targeted anticancer agents were used, a substantial proportion of the 108 drugs that were FDA‐approved and used in the study search. Patients using off‐label targeted therapies were enriched for those with neuro‐oncology diagnoses, highlighting the paucity of effective conventional therapies and FDA‐approved agents with indications for pediatric brain tumors. Most off‐label therapies were discontinued due to progression of disease rather than toxicity.

Our results also showed that 41% of patients receiving off‐label targeted therapy had public insurance, which aligns with population‐based data on insurance coverage for children with cancer. Studies suggest that approximately 30%‐39% of pediatric cancer patients had public insurance at the time of diagnosis,^11^, ^12^ and trial‐based data on children receiving chemotherapy for cancer diagnosis reported 35% were covered only by public insurance.^13^ The slightly higher frequency of public insurance seen in our study may reflect the duration of illness and consequent financial impact on families, as the number of patients with public insurance generally increases over time, either as an addition to their private insurance or as a sole insurance for families who may have experienced financial burden.^14^, ^15^ Due to limitations of our study design, the impact of insurance coverage on access to off‐label targeted therapies was not studied and should be investigated in the future. The comparable frequency of public insurance in our study to that seen in published data suggests that a significant proportion of patients had access to treatment despite potential barriers to access for publicly insured children.

Although 38% of patients met the toxicity definition, only 13% of patients discontinued their off‐label therapy due to toxicity. The toxicity rate in our study is comparable to the rate of dose‐limiting toxicity typically allowed in conventional phase 1 trials. In a previous report of general pediatric patients, 67% of patients were reported to have adverse events with off‐label medication use; however, all of these were grade 1 or 2.^16^ In our study, we found that the dose was often started conservatively below the FDA‐recommended dose and later increased. Given the strong correlation between adult and pediatric maximum tolerated doses,^17^ this conservative approach may explain the low percentage of patients who discontinued treatment as a result of toxicity. A more conservative initial dosing strategy may have been adopted since nearly half of off‐label instances were given with conventional chemotherapy or with other targeted therapy in 23% of cases. Furthermore, many of these patients may have already been pretreated or may not have qualified for clinical trial enrollment due to organ function limitations. It should also be noted that adult patients who begin targeted therapy at the recommended starting dose may require dose de‐escalations, which result in a lower average dose intensity over time than that predicted by the standard starting dose.

While it is difficult to conclude from this study that these off‐label therapies provide long‐term benefit, we found several agents with prolonged duration of use. Of those agents, bevacizumab, sorafenib, and thalidomide had particularly long durations of use in multiple patients. Bevacizumab, the most frequently used off‐label targeted therapy in our study, was used for a median duration of 169 days. Although it is currently indicated for the treatment of recurrent glioblastoma in adults, there is no FDA‐approved indication for pediatric patients diagnosed with central nervous system (CNS) tumors. Several studies have shown its efficacy and safety in pediatric CNS cancers such as relapsed medulloblastoma and low‐grade glioma,^18^, ^19^, ^20^ which may explain the notably high prevalence of its off‐label use in children with CNS tumors.

The common and increasing use of off‐label treatment in pediatric oncology seen in our study also emphasizes the ongoing lack of targeted therapies for children with cancer approved by regulatory agencies. From 2007 to 2017, there have been 78 adult cancer drugs approved by the FDA, yet only 17 (21.8%) drugs received pediatric labeling information.^21^ Since then, the Research to Accelerate Cures and Equity (RACE) for Children Act was passed in 2017 to strengthen the requirements implemented in the Pediatric Research Equity Act (PREA). Under the RACE Act, the FDA will be authorized to mandate evaluation of new therapeutics intended to treat adult cancer if the molecular target is relevant to a pediatric malignancy, and to extend pediatric study requirements for drugs treating rare cancers. Additionally, there have been efforts to reduce the minimum age of eligibility for trials relevant to adolescent cancers from 18 to 12 years, which would expand evaluation of new drugs in a cohort traditionally underrepresented in clinical trials.^21^, ^22^, ^23^, ^24^ It will be important to track the effects of these initiatives on metrics of drug access for minors with cancer, including delays in timing of first‐in‐child trials of oncology agents^25^ as well as on the prevalence of off‐label use in pediatric oncology.

We acknowledge that our study was limited by being a single‐institution study and the results may not be representative of treatment practices at other centers. However, our hospital has relatively high volume and we have surveyed across a decade of patient care. Furthermore, the toxicity data in our study were less comprehensive than those that can be obtained in a prospective trial, as unplanned visits were utilized as a proxy for adverse events. As half of the patients who met toxicity criteria were receiving combination therapy, the limitations of retrospectively analyzing toxicity in combination therapies should be considered. Our measure of toxicity was exclusively utilization‐based and collected by reviewing free text clinical documentation, which can lead to inconsistencies depending on the accuracy and completeness of the documentation. To our knowledge, there is not a standard approach for assessment of toxicities in retrospective or real‐world oncology studies. Further, what may be deemed acceptable toxicity varies across disease groups and disease states, even on prospective early phase trials. Nevertheless, we provide real‐world data on a substantial number of patients that highlight the prevalence of significant adverse events, which may ultimately be most important to clinical practice. Due to limitations of the data captured in our PPIP database, we also had limited comparative data in the control group to determine clinical or patient‐level demographic features associated with off‐label use. We likewise lack data on the extent of molecular testing that may have driven the selection of off‐label therapies, though note that the increasing prevalence of off‐label use parallels the increasing availability of next‐generation sequencing in the field. Finally, as our study involves a comprehensive, yet retrospectively collected heterogeneous set of agents, it was not designed or powered to make conclusions about efficacy of these agents.

In summary, the findings of our study demonstrate that off‐label use of targeted anti‐cancer agents in pediatric oncology is common, has increased in prevalence over the last decade, and involves a wide range of FDA‐approved agents. In particular, this practice is significantly enriched among patients with neuro‐oncology diagnoses, a group of diseases with a paucity of approved agents. Further study is required, particularly among specific genomic subgroups of patients, to determine the safety and efficacy of these medications, some of which may ultimately merit an FDA approval for specific subsets of patients. Such work will be important to ensure that these medications are ultimately used at the correct dose for the appropriate patients.

## DISCLOSURES

SGD has received fees for consulting and advisory board roles from Loxo Oncology and has received travel expenses from Loxo Oncology, Roche, and Salarius. ML, DSS, HR, AL, JC, KB, HA, and CM report no conflicts of interest.

## AUTHOR CONTRIBUTIONS

Mir Lim: Conceptualization, data curation, investigation, methodology, writing—original draft, and writing—review and editing; David S. Shulman: Conceptualization, data curation, investigation, methodology, project administration, writing—original draft, and writing—review and editing; Holly Roberts: Investigation, writing—original draft; Anran Li: Data curation, formal analysis; Jessica Clymer: Writing—review and editing; Kira Bona: Writing—review and editing; Hasan Al‐Sayegh: Data curation, formal analysis, writing—review and editing; Clement Ma: Data curation, formal analysis, writing—review and editing; Steven G. DuBois: Conceptualization, data curation, funding acquisition, investigation, methodology, project administration, supervision, writing—original draft, and writing—review and editing.

## Supporting information

Table S1

Table S2

Table S3

## Data Availability

The data that support the findings of this study are available from the corresponding author upon reasonable request.
