# Supplementary material for: Off‐label prescribing of targeted anticancer therapy at a large pediatric cancer center
Source: Cancer Med. 2020 Aug 4;9(18):6658–66. doi: 10.1002/cam4.3349 (PMC7520353; doi:10.1002/cam4.3349)
Supplement: Supplementary file 1 — Table S1 [file CAM4-9-6658-s001.docx]

**Supplemental Table 1: Targeted anti-cancer agents approved by the FDA between 1997-2017.**

| **Name of off-label drug** | | |
| --- | --- | --- |
| Abarelix | Erlotinib | Ponatinib |
| Abemaciclib | Everolimus | Ra 223 dichloride |
| Abiraterone acetate | Flutamide | Ramucirumab |
| Acalabrutinib | Fulvestrant | Regorafenib |
| Ado-trastuzumab emtansine | Gefitinib | Ribociclib |
| Afatinib | Gemtuzumab ozogamicin | Rituximab |
| Aldesleukin | Goserelin | Romidepsin |
| Alectinib | Histrelin | Rucaparib |
| Alemtuzumab | Ibritumomab tiuxetan | Ruxolitinib |
| Alitretinoin | Ibrutinib | Sonidegib |
| All-trans retinoic acid | Idelalisib | Sorafenib |
| Arsenic trioxide | Imatinib | Sunitinib |
| Atezolizumab | Imiquimod | Talimogene laherparepvec |
| Avelumab | Inotuzumab ozogamicin | Temsirolimus |
| Axicabtagene ciloleucel | Ipilimumab | Thalidomide |
| Axitinib | Ixazomib | Tisagenlecleucel |
| Belinostat | Lanreotide | Toremifene |
| Bevacizumab | Lapatinib | Tositumomab & I-131 |
| Bexarotene | Lenalidomide | Trametinib |
| Bicalutamide | Lenvatinib | Trastuzumab |
| Blinatumomab | Letrozole | Vandetanib |
| Bortezomib | Midostaurin | Vemurafenib |
| Bosutinib | Necitumumab | Venetoclax |
| Brentuximab vedotin | Neratinib | Vismodegib |
| Brigatinib | Nilotinib | Vorinostat |
| Cabozantinib | Niraparib | Ziv-aflibercept |
| Carfilzomib | Nivolumab |  |
| Ceritinib | Obinutuzumab |  |
| Cetuximab | Ofatumumab |  |
| Cobimetinib | Olaparib |  |
| Copanlisib | Olaratumab |  |
| Crizotinib | Omacetaxine mepesuccinate |  |
| Dabrafenib | Osimertinib |  |
| Daratumumab | Palbociclib |  |
| Dasatinib | Panitumumab |  |
| Denileukin diftitox | Panobinostat |  |
| Dinutuximab | Pazopanib |  |
| Durvalumab | Peginterferon alfa-2b |  |
| Elotuzumab | Pembrolizumab |  |
| Enasidenib | Pertuzumab |  |
| Enzalutamide | Pomalidomide |  |
