# Supplementary material for: Off‐label prescribing of targeted anticancer therapy at a large pediatric cancer center
Source: Cancer Med. 2020 Aug 4;9(18):6658–66. doi: 10.1002/cam4.3349 (PMC7520353; doi:10.1002/cam4.3349)
Supplement: Supplementary file 2 — Table S2 [file CAM4-9-6658-s002.docx]

**Supplemental Table 2: Characteristics of off-label agent use**

| **Drug name** | **Number of patients using as off-label** | **Median dose (mg/kg)** | **Median dose (mg/m2)*** | **FDA approved dose (mg/m2)** | **Most common dosing interval** | **Median Duration of Use (days)** | **Frequency of dose modification** | **Frequency of toxicity** |
| --- | --- | --- | --- | --- | --- | --- | --- | --- |
| Afatinib | 1 | -- n=0 | 18 (18, 18) n=1 | 23.53 | Daily | 25 (25, 25) n=1 | 0/1 (0%) | 0/1 (0%) |
| All-trans retinoic acid | 2 | 0.6 (0.4, 0.8) n=2 | 17.8 (12.5, 23) n=2 | 45.00 | Daily, Every 12 Hours | 18 (3, 33) n=2 | 0/1 (0%) | 0/2 (0%) |
| Arsenic trioxide | 1 | 0.2 (0.2, 0.2) n=1 | 4.5 (4.5, 4.5) n=1 | 6.18 | -- | -- n=0 | 0/1 (0%) | 0/1 (0%) |
| Bevacizumab | 156 | 10 (0.5, 18.7) n=160 | 296.1 (105.1, 666.7) n=157 | 411.76 | Every Other Week | 169 (1, 3063) n=161 | 15/160 (9%) | 54/158 (34%) |
| Brentuximab vedotin | 3 | 1.8 (1.8, 1.8) n=3 | 67.3 (55.8, 67.6) n=3 | 74.12 | Every 3 Weeks | 22 (21, 71) n=3 | 0/3 (0%) | 1/3 (33%) |
| Ceritinib | 1 | 18.3 (18.3, 18.3) n=1 | 510 (510, 510) n=1 | 264.71 | Daily | 232 (232, 232) n=1 | 0/1 (0%) | 1/1 (100%) |
| Crizotinib | 3 | 7.2 (6.9, 11.4) n=3 | 265.1 (208, 280) n=3 | 147.06 | Every 12 Hours | 19 (11, 31) n=3 | 1/3 (33%) | 2/3 (67%) |
| Dabrafenib | 5 | 2.5 (1.7, 5.1) n=5 | 75 (64, 146.1) n=5 | 88.24 | Every 12 Hours | 223 (6, 779) n=4 | 4/5 (80%) | 3/5 (60%) |
| Dasatinib | 23 | 2 (0.9, 61) n=23 | 60 (36.9, 80.6) n=22 | 58.82 | Daily | 84.5 (15, 928) n=20 | 3/22 (14%) | 9/21 (43%) |
| Dinutuximab | 9 | 0.8 (0.5, 0.8) n=9 | 17.5 (17.5, 17.5) n=9 | 17.50 | Other | 25 (4, 270) n=9 | 2/9 (22%) | 6/9 (67%) |
| Erlotinib | 11 | 2.4 (1.6, 6) n=10 | 71.5 (60, 145.1) n=10 | 58.82 | Daily | 35 (1, 245) n=9 | 2/9 (22%) | 1/9 (11%) |
| Everolimus | 38 | 0.2 (0.1, 0.2) n=34 | 5 (2.3, 5.9) n=34 | 4.50 | Daily | 161 (25, 1145) n=36 | 11/38 (29%) | 10/36 (28%) |
| Imatinib | 10 | 11.2 (3.9, 333) n=10 | 325.7 (175.6, 373) n=10 | 340.00 | Daily | 78 (28, 3412) n=9 | 6/10 (60%) | 4/9 (44%) |
| Imiquimod | 1 | -- n=0 | -- n=0 | . | Other | 96 (96, 96) n=1 | 0/1 (0%) | 0/1 (0%) |
| Ipilimumab | 5 | 1 (1, 3) n=5 | 32.7 (27, 79.2) n=5 | 123.53 | Every 3 Weeks | 21 (1, 146) n=5 | 0/5 (0%) | 1/5 (20%) |
| Lapatinib | 1 | 20.8 (20.8, 20.8) n=1 | 735.3 (735.3, 735.3) n=1 | 735.29 | Daily | 88 (88, 88) n=1 | 0/1 (0%) | 0/1 (0%) |
| Lenalidomide | 1 | 1.6 (1.6, 1.6) n=1 | 68 (68, 68) n=1 | 5.88 | Daily | 72 (72, 72) n=1 | 0/1 (0%) | 0/1 (0%) |
| Nilotinib | 1 | 5.8 (5.8, 5.8) n=1 | 214 (214, 214) n=1 | 230.00 | Every 12 Hours | -- n=0 | 0/1 (0%) | 0/1 (0%) |
| Nivolumab | 6 | 3 (1, 3) n=6 | 83.8 (27, 130) n=6 | 123.53 | Every 3 Weeks, Every Other Week | 29 (1, 618) n=6 | 0/6 (0%) | 2/6 (33%) |
| Olaparib | 2 | 3.8 (3.2, 4.4) n=2 | 108 (71, 145) n=2 | 235.29 | Daily, Other | 104 (1, 207) n=2 | 1/2 (50%) | 1/2 (50%) |
| Olaratumab | 1 | 15 (15, 15) n=1 | 560.6 (560.6, 560.6) n=1 | 617.65 | Other | 8 (8, 8) n=1 | 0/1 (0%) | 1/1 (100%) |
| Palbociclib | 1 | 2.7 (2.7, 2.7) n=1 | 75 (75, 75) n=1 | 73.53 | Other | 66 (66, 66) n=1 | 0/1 (0%) | 1/1 (100%) |
| Panobinostat | 4 | 0.9 (0.6, 1.1) n=4 | 24 (19.2, 26) n=4 | 11.76 | Other | 25.5 (1, 233) n=4 | 0/3 (0%) | 0/3 (0%) |
| Pazopanib | 28 | 9.5 (3.4, 19.5) n=27 | 367.1 (73.1, 488.5) n=28 | 470.59 | Daily | 60 (15, 586) n=25 | 15/28 (54%) | 9/28 (32%) |
| Pembrolizumab | 17 | 2 (2, 2.5) n=17 | 69 (46.4, 105) n=17 | 82.35 | Every 3 Weeks | 28 (1, 1240) n=17 | 1/17 (6%) | 5/17 (29%) |
| Pomalidomide | 1 | 0.1 (0.1, 0.1) n=1 | 2.4 (2.4, 2.4) n=1 | 2.35 | Other | 29 (29, 29) n=1 | 0/1 (0%) | 0/1 (0%) |
| Ponatinib | 1 | 1 (1, 1) n=1 | 22.4 (22.4, 22.4) n=1 | 26.47 | Daily | 31 (31, 31) n=1 | 0/1 (0%) | 1/1 (100%) |
| Regorafenib | 3 | 2 (1.3, 2.5) n=3 | 70.2 (57.4, 89.7) n=3 | 70.59 | Other | 19 (10, 41) n=3 | 0/3 (0%) | 0/3 (0%) |
| Rituximab | 59 | 11 (8.3, 17.7) n=59 | 375 (375, 378) n=59 | 375.00 | Other | 91 (1, 443) n=57 | 1/59 (2%) | 20/58 (34%) |
| Romidepsin | 1 | 0.4 (0.4, 0.4) n=1 | 14 (14, 14) n=1 | 14.00 | Other | 54 (54, 54) n=1 | 0/1 (0%) | 0/1 (0%) |
| Sonidegib | 3 | 25.2 (19, 28.7) n=3 | 680 (509.4, 720.7) n=3 | 117.65 | Daily | 93 (24, 494) n=3 | 0/3 (0%) | 1/3 (33%) |
| Sorafenib | 52 | 4.1 (1.6, 10.4) n=50 | 117 (80, 313) n=51 | 235.29 | Every 12 Hours | 104 (1, 559) n=53 | 23/54 (43%) | 23/52 (44%) |
| Sunitinib | 4 | 0.5 (0.4, 0.7) n=4 | 19.3 (13.8, 21.7) n=4 | 29.41 | Other | 5 (1, 477) n=4 | 0/4 (0%) | 1/4 (25%) |
| Temsirolimus | 25 | 2.1 (0.4, 3.5) n=25 | 73.1 (15, 76) n=25 | 14.71 | Weekly | 209 (1, 1891) n=24 | 2/25 (8%) | 9/25 (36%) |
| Thalidomide | 59 | 3 (1.2, 10) n=59 | 88.6 (28.8, 263) n=59 | 117.65 | Daily | 113 (6, 1201) n=54 | 37/58 (64%) | 30/56 (54%) |
| Trametinib | 17 | 0.03 (0.02, 0.1) n=16 | 0.8 (0.3, 1.5) n=16 | 1.18 | Daily | 90 (17, 826) n=15 | 5/17 (29%) | 8/17 (47%) |
| Vismodegib | 1 | -- n=0 | -- n=0 | -- | -- | 223 (223, 223) n=1 | 1/1 (100%) | 0/1 (0%) |
| Vorinostat | 5 | 6.8 (6.3, 10.8) n=5 | 210 (185, 266.7) n=5 | 235.29 | Daily | 69 (1, 102) n=5 | 2/5 (40%) | 4/5 (80%) |

*In instances in which dosing was based on weight or a fixed dose, mg/m2 was computed based on a BSA of 1.7.
