# Supplementary material for: Off‐label prescribing of targeted anticancer therapy at a large pediatric cancer center
Source: Cancer Med. 2020 Aug 4;9(18):6658–66. doi: 10.1002/cam4.3349 (PMC7520353; doi:10.1002/cam4.3349)
Supplement: Supplementary file 3 — Table S3 [file CAM4-9-6658-s003.docx]

**Supplemental Table 3**. Details of 20 patients with complete response to off-label therapies given without concomitant cytotoxic chemotherapy.

| **Diagnosis** | **Off-Label Regimen** |
| --- | --- |
| Post-transplant lymphoproliferative disease (n=4) | Rituximab |
| Chronic myelogenous leukemia (n=2) | Dasatinib |
| Acute myeloid leukemia | Sorafenib |
| Acute promyelocytic leukemia | All-trans retinoic acid and arsenic trioxide |
| Basal cell carcinoma | Vismodegib |
| Basal cell carcinoma | Imiquimod |
| B-cell acute lymphocytic leukemia | Dasatinib |
| B-cell acute lymphocytic leukemia | Imatinib |
| Chronic myelogenous leukemia | Imatinib |
| Ependymoma | Vorinostat |
| High-grade glioma | Dabrafenib and trametinib |
| Low-grade glioma | Dabrafenib |
| Optic pathway glioma | Everolimus |
| Osteosarcoma | Sorafenib |
| Rhabdomyosarcoma | Temsirolimus |
| Sarcoma, NOS | Ceritinib |
